# Mapping the landscape of women entrepreneurs in micro and small enterprises: Trends, themes, and insights through systematic review and text mining

**DOI:** 10.12688/f1000research.163030.1

**Published:** 2025-04-10

**Authors:** Thanh Binh Nguyen, Mohan Kumar, Deepak Kumar Sahoo, Neha Nain, Mohit Yadav, Vartika Dadhich

**Affiliations:** 1 Faculty of Business Administration, Banking Academy, Hanoi, Vietnam; 2 Faculty of Commerce & Management, SGT University, Gurugram, Haryana, India; 3 Institute of Management & Information Science, Bhubaneswar, India; 4 The Technological Institute of Textile and Sciences, Bhiwani, India; 5 OP Jindal Global University, Sonipat, Haryana, India; 6 T.A. Pai Management Institute, Bengaluru, Manipal Academy of Higher Education, Manipal, Karnataka, India

**Keywords:** Women Entrepreneurs, Micro and Small Enterprises, Economic Development, Systematic Literature Review, Latent Dirichlet Allocation

## Abstract

**Background:**

This study explores the pivotal role of women entrepreneurs in fostering economic growth and social transformation, focusing on micro and small enterprises (MSEs). Despite the significant rise in women entrepreneurship, challenges persist, including gender-based barriers, limited access to capital, and regional disparities. Understanding these dynamics is essential for formulating policies that support and empower women entrepreneurs.

**Method:**

The present study employed a systematic literature review (SLR) and Latent Dirichlet Allocation (LDA), an advanced topic modeling technique, to identify prevalent themes within the literature. Data was extracted from Scopus-indexed journals (2000–2024), systematically filtered, and analyzed to uncover key patterns in women entrepreneurship research.

**Results:**

The analysis identifies four central themes: (1) Women in Entrepreneurship and Gender Roles, (2) Small Business and Women Enterprises, (3) Social Impact of Women Entrepreneurs, and (4) Challenges Faced by Female Entrepreneurs. The findings highlight the transformative potential of digital platforms in enhancing business opportunities for women and addressing traditional constraints, such as mobility and market access.

**Conclusion:**

This study highlights the need for targeted interventions to enhance digital literacy, bridge socio-cultural disparities, and establishing inclusive policies that foster a supportive entrepreneurial ecosystem. By synthesizing existing literature, this research provides actionable recommendations for policymakers, financial institutions, and stakeholders to strengthen women-led enterprises and drive gender-equitable growth.

## 1. Introduction

Women’s entrepreneurship is a crucial catalyst for economic growth and social advancement, particularly in developing countries. In recent decades, there has been a significant rise in the number of women engaging in entrepreneurship both worldwide and in India. This transition is ascribed to multiple variables, such as policy endorsement, expanding educational prospects, and evolving societal perceptions of women’s involvement in the labor market. Despite these achievements, women entrepreneurs (WEs) continue to encounter substantial obstacles, including cultural and institutional limitations as well as restricted access to financial resources and technology.

Although women constitute approximately 50% of the population, only 13.76% of proprietary firms are owned or managed by them (All India Report of Sixth Economic Census). Moreover, WEs in India account for only 10.24% of the employment prospects across several economic activities. These numbers indicate a notable gender disparity in entrepreneurial engagement, highlighting the need for focused initiatives to advance gender equity in this field. Promisingly, the trends suggest advancements in specific domains. The proportion of WEs in urban areas has risen to 34.8% in the 6th Economic Census, an increase from 25.9% in the 5th Census. Correspondingly, women’s involvement in agricultural entrepreneurship has increased from 15.7% to 34.3%. Nonetheless, obstacles remain.

Women-led enterprises are primarily small-scale, frequently functioning without employed personnel or specialized infrastructure. They are focused on conventional areas such as tailoring, healthcare, education, and arts and crafts, indicating restricted diversification ( Shuvam & Mohanty, 2023; Kumar, 2021). Regional inequalities exacerbate the complexity of the landscape. States such as Tamil Nadu, Kerala, and West Bengal excel in women-led firms, whereas the North-Eastern states, despite their matrilineal cultures, demonstrate low participation rates. This disparity underscores the influence of socio-cultural norms, economic development, and legislative frameworks on women’s entrepreneurial endeavours. The Government of India has implemented various policies and efforts to promote women entrepreneurship. The Prime Minister’s Employment Generation Program (PMEGP) allocates 30% of its funding to small and micro firms operated by women (Women Entrepreneurs|Ministry of Micro, Small & Medium Enterprises). These initiatives seek to empower women entrepreneurs through the provision of financial assistance, training, and mentorship. Nonetheless, the efficacy of these programs is inconsistent, necessitating further research to comprehend their effects.

### 1.1 Success Narratives: the role of role models in women entrepreneurship

The success stories of Indian women entrepreneurs offer powerful narratives of resilience and empowerment. For example, the entrepreneurial journey of Falguni Nayar, founder of Nykaa, exemplifies how women can break conventional barriers and lead successful businesses in male-dominated industries. Nykaa’s rapid growth in the beauty sector not only demonstrates the entrepreneurial spirit of Indian women but also highlights the transformative capacity of women-led businesses in India. Nayar’s success story underscores the potential for women to establish scalable and impactful businesses, which, in turn, inspire future generations of women entrepreneurs. These success stories play a critical role in challenging stereotypes and fostering a more inclusive entrepreneurial ecosystem in India. They exemplify how supportive frameworks, such as government policies, financial access, and educational opportunities, contribute to the growth and sustainability of women-owned enterprises ( Kumar et al., 2023; Malhan et al., 2021; Tabassum & Nayak, 2021). Moreover, they demonstrate the resilience of women entrepreneurs who, despite facing gendered challenges, are able to innovate and lead successful ventures that contribute significantly to the economy.

This study aims to address the knowledge gap by performing a systematic literature review (SLR) of current research on women’s entrepreneurship in India. This research seeks to deliver a thorough overview of the problems and opportunities encountered by WEs in India through the analysis of empirical studies and the synthesis of relevant results. The study also emphasises geographical disparities and policy ramifications, providing significant insights for scholars, policymakers, and practitioners.

## 2. Literature review

The studies explored the multifaceted challenges and opportunities faced by women entrepreneurs, with much of the literature focusing on key aspects such as work-life balance ( Agarwal & Lenka, 2015), gender dynamics in entrepreneurial behavior ( Marlow, 2014) and the well-being of entrepreneurs ( White & Gupta, 2020). While these studies provide valuable insights into individual challenges, they often lack a comprehensive approach that considers the complex interplay between policy, socio-cultural factors, and entrepreneurial performance in the Indian context. This gap becomes particularly evident when examining women entrepreneurship in the unique socio-economic landscape of India, where regional differences and varying access to resources significantly affect entrepreneurial outcomes ( Bastian et al., 2018; Panda, 2018).

Although existing studies acknowledge the gendered nature of entrepreneurship there is a need for research that integrates the broader socio-political factors that shape the entrepreneurial ecosystem in India ( Agarwal & Lenka, 2015). Few studies have examined the combined effects of government policies, cultural norms, and entrepreneurial performance in a single analytical framework. As such, there remains a significant gap in understanding how these factors intersect to either facilitate or hinder the success of women-led businesses in India.

### 2.1 Determinants affecting women entrepreneurs in MSMEs

Female entrepreneurs encounter a variety of hurdles and possibilities that profoundly impact their performance. Empirical research from many regions identifies shared drivers including educational attainment, financial access, market possibilities, regulatory impediments, infrastructure, and cultural norms ( Abiodun & Amos, 2018; Mozumdar et al., 2020; Welsh et al., 2018).

In India, these elements frequently intersect with socio-cultural dynamics, intensifying barriers for women entrepreneurs. Restricted access to capital persists as a substantial obstacle, since numerous women entrepreneurs encounter difficulties in obtaining loans due to entrenched gender biases inside financial institutions. Likewise, insufficient access to markets and technology further constrains their economic expansion. Digital transformation has become a pivotal factor, allowing WEs to surmount conventional obstacles via online platforms. Research indicates that digital transformation and online platforms substantially benefit women’s MSMEs by facilitating access to funding, training, and networks, hence promoting resilience and innovation ( Ahl, 2006; Poggesi et al., 2020; White & Gupta, 2020). The significance and effects of these characteristics differ across geographical and economic situations, requiring customized responses.

### 2.2 Geographical and situational disparities

The socio-economic variety of India fosters a distinctive entrepreneurial environment. States such as Tamil Nadu, Kerala, and Karnataka have a greater share of women-led firms attributable to progressive legislation and enhanced access to education and resources. In contrast, states in the North-East, despite their matrilineal frameworks, exhibit diminished entrepreneurial engagement among women. This contradiction highlights the necessity for context-specific study to comprehend the interaction between cultural norms and entrepreneurial results ( Shuvam & Mohanty, 2023).

Despite the abundance of global studies on women’s entrepreneurship, there is a notable deficiency of region-specific research, especially in developing economies such as India ( Bastian et al., 2018; Panda, 2018). Women-led firms in India encounter distinct hurdles arising from cultural norms, restricted access to resources, and insufficient policy enforcement ( Agarwal & Lenka, 2018). Notwithstanding these limitations, positive indicators of expansion in urban and agricultural sectors suggest the emergence of new entrepreneurial dynamics (All India Report of Sixth Economic Census).

These discrepancies also indicate disparities in policy execution. Urban regions exhibit elevated involvement rates among female entrepreneurs, partially attributable to enhanced access to infrastructure and markets. Nevertheless, rural regions are deficient, underscoring the necessity for focused initiatives to rectify regional inequalities.

### 2.3 Digitalization and its effects

Digital platforms have profoundly altered the entrepreneurial landscape for women. Through the provision of training, mentorship, and financial resources, these platforms empower women entrepreneurs to cultivate creative company models and access wider markets. Digital entrepreneurship has enabled women to overcome conventional obstacles, like restricted mobility and limited access to networks ( Shakeel et al., 2020). The amalgamation of digital marketing and e-commerce methods has augmented the competitiveness of women-led firms, especially in areas such as arts and crafts, education, and health.

India’s diverse socio-cultural and economic landscape presents both obstacles and opportunities for women entrepreneurs. Regional variations, such as caste, religion, and geographical location, significantly impact entrepreneurial performance and outcomes. For example, women entrepreneurs in southern states like Kerala and Tamil Nadu benefit from better infrastructure and government support, leading to more successful ventures. In contrast, women in northern and eastern states, such as Uttar Pradesh and Bihar, often face greater barriers, including limited access to education, capital, and markets ( Bastian et al., 2018; Panda, 2018). These disparities highlight the importance of crafting region-specific policies and interventions that address the unique challenges women face across India. The studies on women entrepreneurship in India have provided valuable insights into the challenges and motivations of women business owners, there is a clear need for more holistic research that considers the complex interplay of gender, culture, and entrepreneurial outcomes ( Mohan Kumar, Yadav & Sahoo, 2025). The existing literature, though insightful, often fails to address the unique socio-cultural factors that influence the entrepreneurial journey of women entrepreneurs. As the Indian ecosystem continues to evolve, there is a pressing need for research that can integrate these factors and contribute to more tailored policy interventions that foster the growth of women-led enterprises.

In this research paper, the authors aim to explore the following research questions:

**RQ1.** What are the annual publication trends in the domain of women entrepreneurship? (Manual and using LDA).

**RQ2.** What are the key themes in the women entrepreneurship domain? (Using LDA).

**RQ3**. What are the most prominent keywords in the said domain? (Using LDA-Python).

## 3. Why systematic literature review in this domain?

A comprehensive literature evaluation is necessary to consolidate findings and pinpoint research gaps due to the fragmented nature of current research. Systematic Literature Reviews (SLRs) offer a methodical framework for examining trends, difficulties, and opportunities within a research field. A literature review provides historical perspective and informs future research in a certain topic ( Tranfield et al., 2003). A systematic literature review, or “structured” review, is widely used in management research to describe, locate, evaluate, and interpret existing research on a topic. This study utilizes the 4W (What, Where, How, Why) paradigm, renowned for its efficacy in management research, to deliver a thorough analysis of women entrepreneurship in India ( Callahan, 2014; Lim et al., 2021). Current literature on WEs primarily emphasizes particular topics, including gender dynamics, work-life balance, and regional contexts ( Jennings & Brush, 2013; Rashid & Ratten, 2020). Limited studies offer a thorough summary of research on Indian WEs. A systematic literature review addressing this gap is crucial for identifying research trends, elucidating problems, and guiding future inquiries.

This study focuses on research about women entrepreneurs (WEs) operating within India, excluding studies on migrant women entrepreneurs. For defining WEs, the study adopted the most inclusive definition: “women or groups of women who initiate, organize, and operate a business enterprise” ( Hemalatha & Senthil Nayaki, 2014). This broader definition contrasts with the restrictive Government of India’s criteria, which require at least 51% ownership by women and 51% employment generated for women. Given that 83% of Indian WEs operate without additional hired workers (All India Report of Sixth Economic Census), the broader definition ensures the inclusion of a significant portion of relevant research.

## 4. Methods

A literature search was performed for studies containing keywords including (“women entrepreneurs” OR “female entrepreneurs” OR “female-owned enterprises” OR “women-led enterprises” OR “women-owned businesses” OR “female entrepreneurship” OR “women entrepreneurship”) AND (“micro enterprises” OR “small enterprises” OR “micro and small enterprises” OR “MSEs” OR “MSMEs” OR “small businesses”) to search titles, keywords, and abstracts in the Scopus database. This study’s search process, covering the years 2000 to 2024, was meticulously structured to select the most pertinent and high-caliber papers on women’s entrepreneurship and micro and small businesses (MSEs). A first search across prominent academic publications produced a substantial collection of 514 articles. A series of meticulously applied filters were utilized to enhance these outcomes. The focus topics were refined to Business, Management and Accounting, Economics, Econometrics and Finance, and Social Sciences, resulting in a total of 488 articles. The document type was then limited to Articles, omitting forms such as book chapters, conference proceedings, and reviews, so reducing the total to 377 articles. The language filter was configured to English to maintain consistency and accessibility, resulting in a total of 375 articles. Subsequently, the source type was restricted to journals, omitting trade publications and other non-scholarly materials, resulting in 372 articles. The publication step was subsequently limited to Final, ensuring that only validated and completed research was incorporated, hence limiting the total to 367 articles. Ultimately, to enhance accessibility and transparency, the selection was narrowed to encompass solely Open Access papers, yielding a total of 107 articles.

The titles and abstracts of each of these 107 papers were then carefully examined to ascertain their applicability to the study’s emphasis on MSEs and women’s entrepreneurship. During this stage, 55 papers that did not conform to the research objectives or pertained to peripheral themes were excluded. This meticulous method culminated in a final dataset of 52 articles considered directly pertinent and appropriate for comprehensive study. This rigorous multi-stage process ensured that the chosen papers were of high academic quality, accessible, and clearly matched with the research aims, thereby establishing a solid platform for significant insights into women’s entrepreneurship and micro and small enterprises (MSEs).

Data extraction was performed by qualitative content analysis. A grid was constructed to document essential information from the chosen papers, encompassing title, author(s), journal, year of publication, methodology (quantitative/qualitative/mixed), data collection method (survey/interview), sample size, geographic focus (specific regions), theoretical framework, objectives, findings, industry (manufacturing/service), sector (organized/unorganized), and location (urban/rural).

The results and discussions were structured using the 4W (What, Where, How, Why) review format, as adopted by ( Billore & Anisimova, 2021) which was suggested by ( Callahan, 2014). This format allowed for systematic presentation and detailed analysis of themes, providing a comprehensive understanding of the literature on WEs in India.

**Figure 1.**
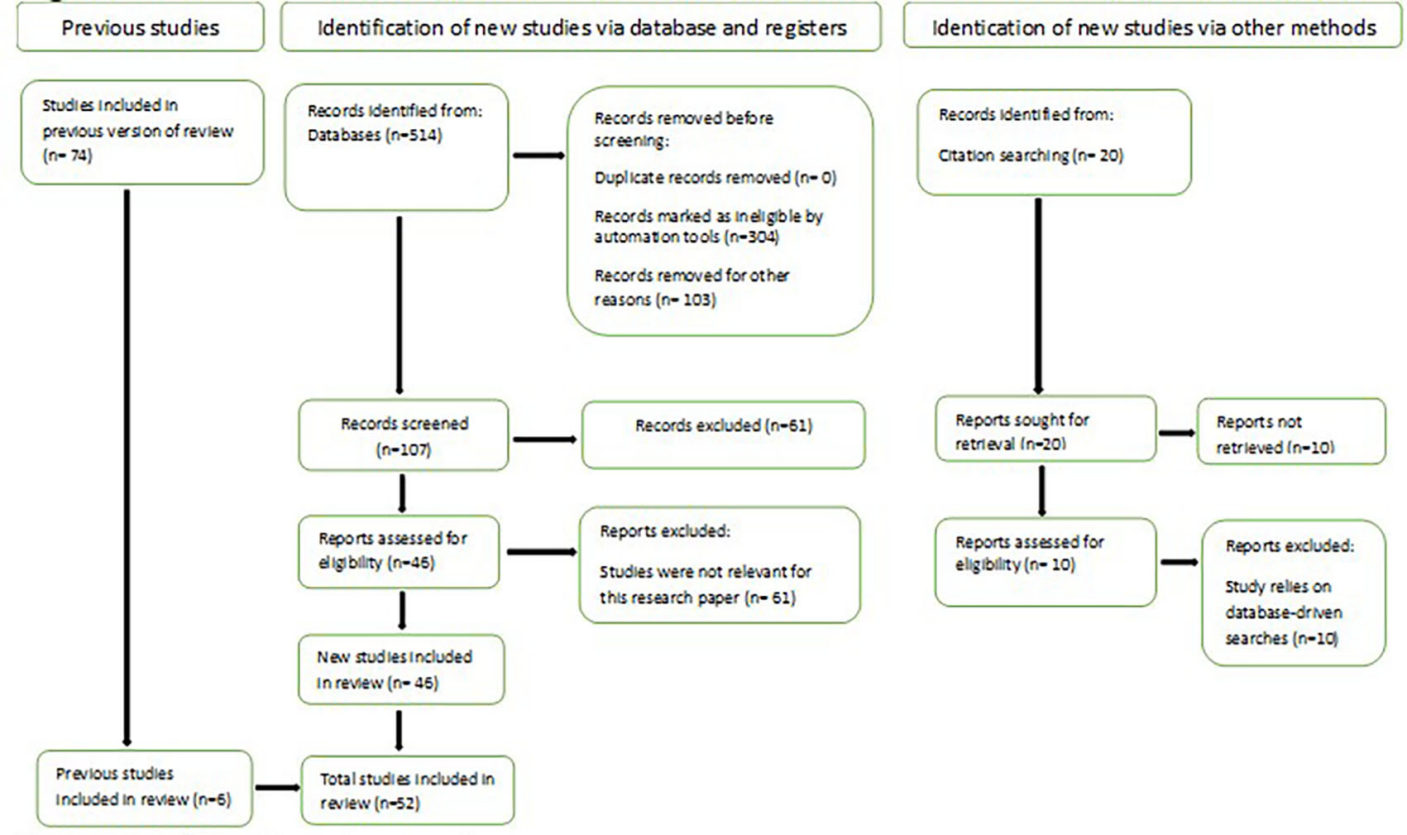
PRISMA Guidelines. Source: Authors Development.

Moher et al. (2009) introduce PRISMA, which stands for Preferred Reporting Items for Systematic Reviews and Meta-Analyses is a framework designed to guide researchers in conducting and reporting systematic reviews and meta-analyses transparently and comprehensively. It is commonly used in various disciplines, particularly in health sciences and social sciences, to ensure clarity and quality in the review process. The process begins with 74 studies from a previous study out of which 6 previous studies are included in review. New studies were identified through database searches, yielding 514 records. After removing 304 ineligible records via automation tools and 103 records for other reasons, 107 records were screened. Of these, 61 were excluded for irrelevance, leaving 46 reports assessed for eligibility. All 46 reports met the criteria and were included in the final review. Additionally, 20 studies were identified through citation searching, but 10 were not retrieved. The remaining 10 were assessed, but all were excluded as they relied on database-driven searches. Ultimately, the review included a total of 52 studies, combining both previously included and newly identified studies as reflected in Figure 1. This structured approach ensures transparency, minimizes bias, and enhances the reproducibility of systematic reviews.

### 4.1 Topic modeling

To develop a better understanding of the prominent themes in the 52 articles on Women Entrepreneurs in Micro and Small Enterprises, topic modeling was used to the most commonly used phrases in the compilation. To attain this objective, topic modeling ( Nikolenko et al., 2017) was applied to the articles. Topic modeling is an unsupervised machine-learning technique that does not rely on a predefined list of tags or training data produced by humans. We utilized the Genism Python framework for vector space topic modeling ( Řehůřek & Sojka, 2011), which uses the Latent Dirichlet allocation (LDA) algorithm ( Blei et al., 2003) to map each article into a set of topics and a set of words for a particular subject.

$$\left(W,Z,\theta ,⊘;\alpha ,\beta \right)=\prod_{i=1}^{M}P\left(\theta_{i};\alpha \right)\prod_{i=1}^{K}P\left(⊘;\beta \right)\prod_{i=1}^{N}P\left(Z_{j,i}|\theta_{i}\right)P\left(W_{j,i}⊘z_{j,i}\right)$$

In this equation, α and β represent Dirichlet distributions, θ and ∅ represent multinomial distributions, and Z is the vector containing all word themes across all articles. W is the vector containing all words from all documents. M number of documents, K number of topics and N number of words. To determine the appropriate subject counts for our 52 articles, we employed two minimum themes and ten maximum topics.

The analysis of the selected studies on women entrepreneurship and micro and small enterprises (MSEs) using Latent Dirichlet Allocation (LDA) revealed several key themes, which were grouped into four main categories based on common core ideas ( Mo et al., 2015). The themes identified include Women in Entrepreneurship and Gender Roles, Small Business and Women Enterprises, Social Impact of Women Entrepreneurs, and Challenges Faced by Female Entrepreneurs. In this study, Latent Dirichlet Allocation (LDA) was applied using the Genism Python framework to identify key themes in women entrepreneurship research. Preprocessing steps included tokenization, stop-word removal, stemming, lowercasing, and lemmatization to refine textual data. The hyperparameters were set at α = 0.1 and β = 0.01 to ensure optimal topic sparsity. The coherence score method was used to determine the optimal number of topics, ranging from 2 to 10, based on word co-occurrence patterns. These methodological refinements enhanced topic interpretability, ensuring a systematic and data-driven approach to analyzing trends in women-led micro and small enterprises (MSEs).

**Figure 2.**
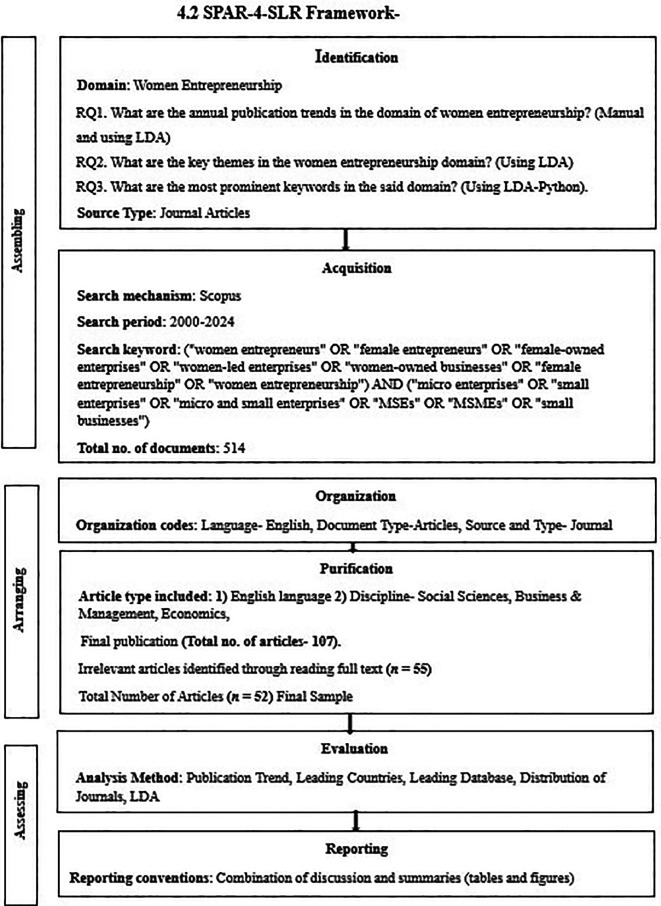
SPAR-4-SLR Framework. Source: Author’s own development.

The identified themes align with existing research exploring gender and entrepreneurship in small businesses and micro enterprises, especially within the context of women-led enterprises. This study, using LDA, contributes to the growing body of literature on women entrepreneurship by offering a data-driven approach to identifying themes within the field ( Figure 2). The flexibility of LDA in analyzing large textual datasets enabled a deeper understanding of the structural issues and opportunities faced by women entrepreneurs in micro and small enterprises ( Hechavarria et al., 2019; Tegegne et al., 2024). These findings also echo the work of Molina-Azorín et al., (2012), who highlighted the efficacy of mixed-methods approaches in entrepreneurship research to capture the complex and dynamic nature of women-led businesses.

## 5. Data analysis

### 5.1 Annual scientific production

**Figure 3.**
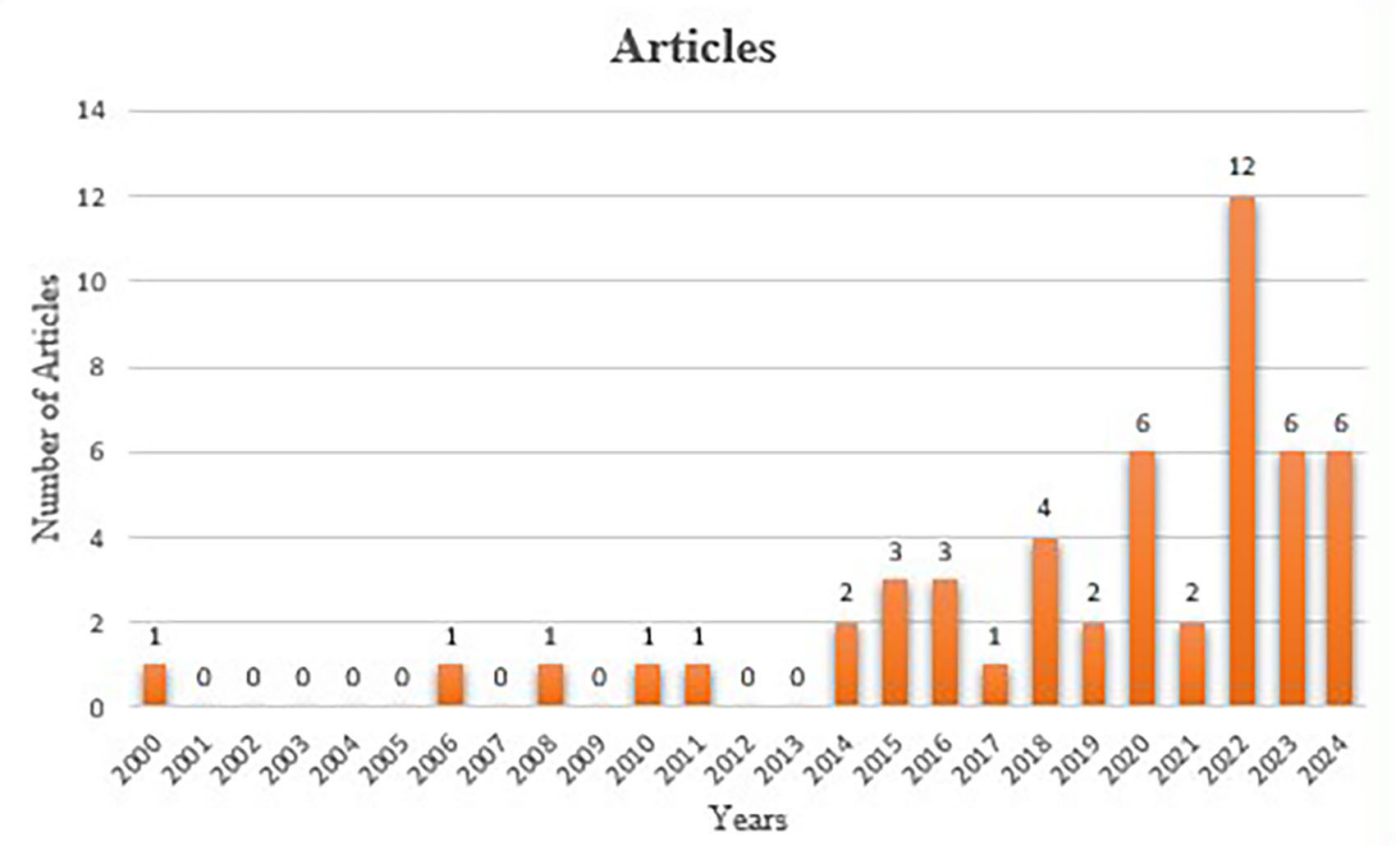
Annual Scientific Production. Source: Author’s own development.

The bar graph depicts annual scientific production from 2000 to 2024 as assessed by the number of papers published each year. The early years, from 2000 to 2004, exhibit negligible productivity, with either no articles or a single article produced each year. From 2005 to 2013, the productivity was consistently low, with sporadic years yielding only one article. A steady rise in scientific output is noted from 2014, commencing with two papers in that year and culminating in four articles in 2017. Between 2018 and 2020, production exhibited minor fluctuations. A major surge happens in 2021, with the publication of 12 papers, the highest output throughout this time span. The output levels out between 2022 and 2024, with six articles released annually. The graph illustrates a gradual onset in the early 2000s, succeeded by consistent growth in subsequent years, culminating in a peak in 2021 and maintained productivity thereafter clearly visible in Figure 3.

### 5.2 Year-wise distribution of lda-derived themes on women entrepreneurship (2000–2024)

**Figure 4.**
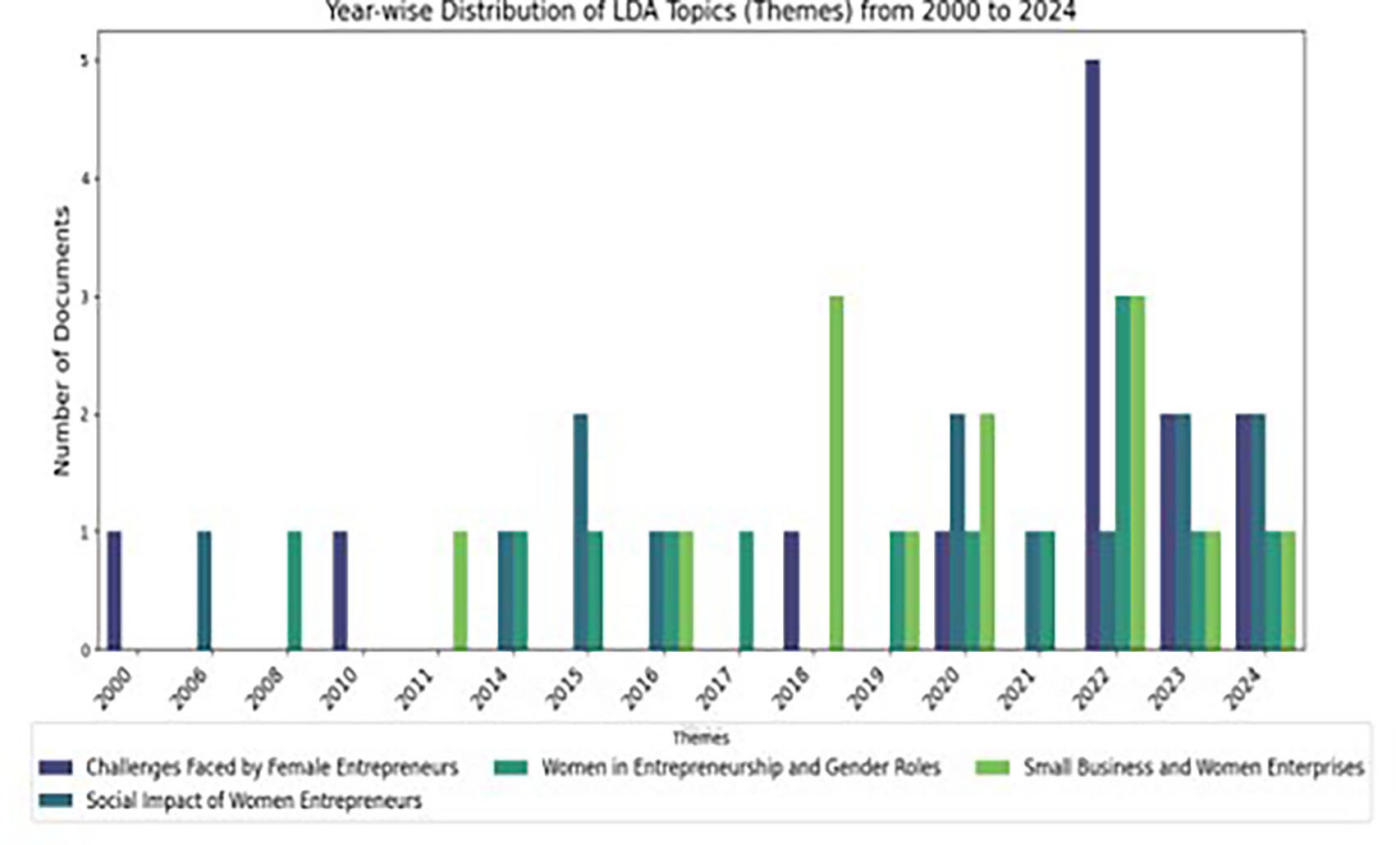
Year-wise Distribution. Source: Author’s own development.

The bar graph represented by Figure 4 depicts the annual distribution of four LDA-derived topics concerning women entrepreneurship from 2000 to 2024: Challenges Faced by Female Entrepreneurs, Women in Entrepreneurship and Gender Roles, Small Business and Women Enterprises, and Social Impact of Women Entrepreneurs. From 2000 to 2014, minimal research effort is evident, with merely one document covering an issue annually. Following 2015, there is a notable increase in research interest, with substantial peaks observed in 2018, 2022, and 2023. The challenges encountered by female entrepreneurs reached a zenith in 2022, with five papers representing the highest frequency among all themes, but small business and women enterprises exhibited significant activity in 2018 and 2022. Themes include the Social Impact of Women Entrepreneurs and Women in Entrepreneurship and Gender Roles exhibit continuous representation, with notable surges in 2020 and 2023. The graph highlights an increasing emphasis on women’s entrepreneurship, propelled by worldwide initiatives for gender equality and economic inclusion.

### 5.3 Key themes in women’s entrepreneurship identified through lda topic modeling

**Figure 5.**
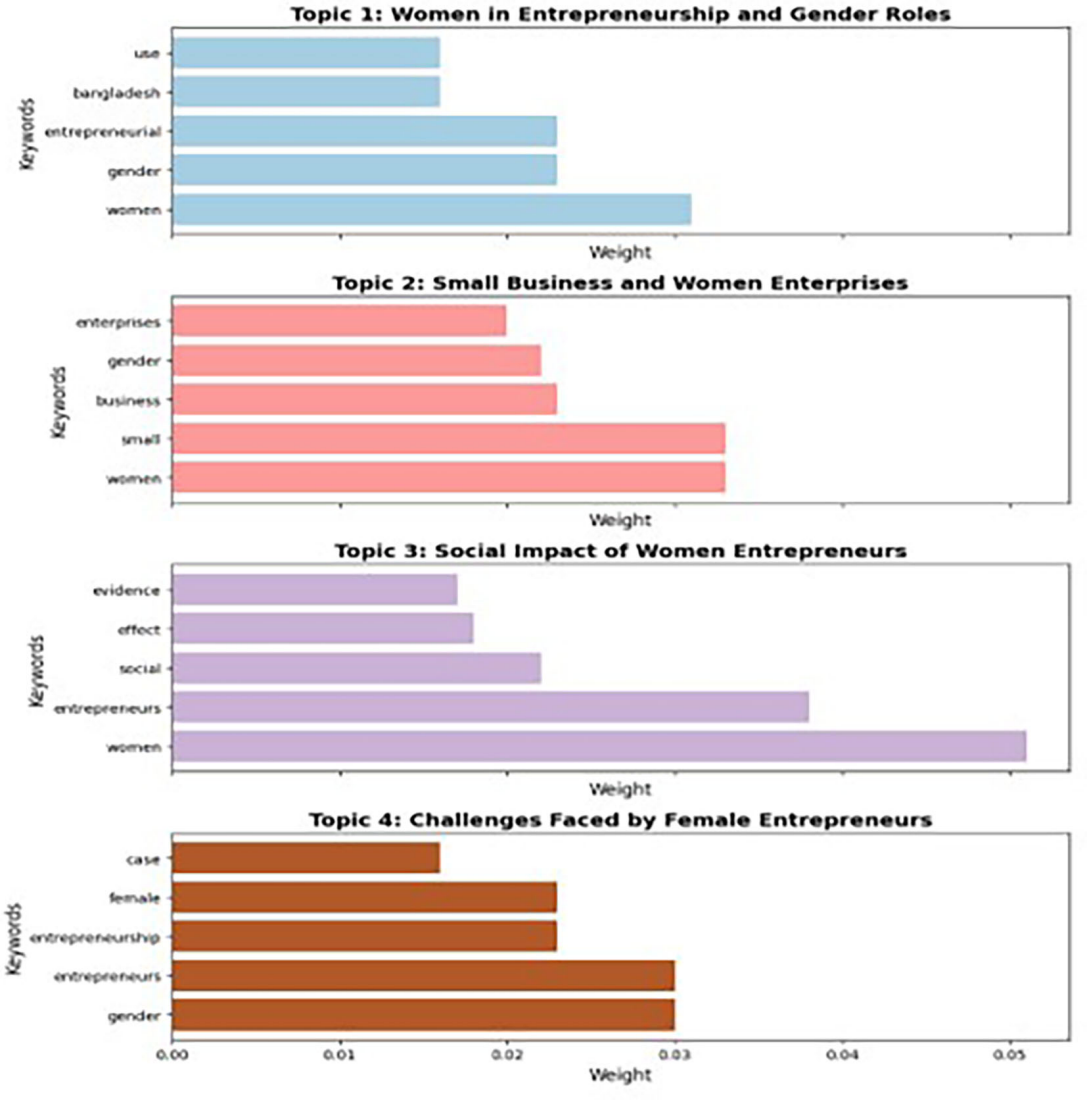
Key Themes. Source: Author’s own development.

As reflected in Figure 5. a Latent Dirichlet Allocation (LDA) study yielded four major themes in women’s entrepreneurship, which are shown in the figure. Topic 1, Women in Entrepreneurship and Gender Roles, explores the influence of gender on entrepreneurship within specific regional contexts, such as Bangladesh. Topic 2, Small Business and Women Enterprises, examines women’s participation in small-scale enterprises and gender dynamics. Topic 3, Social Impact of Women Entrepreneurs, emphasizes their contributions to societal transformation and community advancement. Topic 4, Challenges Encountered by Female Entrepreneurs, examines gender-specific obstacles using case studies. This analysis presents a systematic examination of topics, yielding insights into gender, entrepreneurship, societal impact, and obstacles, thereby impacting research and policy.

**Theme 1: Women in entrepreneurship and gender roles**

This theme emphasizes the distinctive experiences and roles of women in entrepreneurial endeavours, concentrating on the relationship between gender and entrepreneurship. The use of keywords such as “women,” “gender,” and “entrepreneurial” indicates that these subject highlights gender-specific elements affecting women’s involvement in entrepreneurship. The mention of “Bangladesh” suggests a geographical emphasis, possibly examining how the cultural, social, or economic circumstances in nations such as Bangladesh influence the entrepreneurial environment for women. This theme examines the impact of entrepreneurship on the economic and social empowerment of women. Research indicates that women entrepreneurs get enhanced financial autonomy, superior decision-making authority, and elevated societal acknowledgement ( Liao et al., 2022).

**Theme 2: Small business and women enterprises**

This theme centers around women’s involvement in small businesses and enterprises. Keywords such as “small”, “business”, “women”, and “enterprises” highlight the emphasis on women entrepreneurs operating in small-scale or grassroots-level businesses. The second theme examines how women entrepreneurs contribute to micro and small enterprises (MSEs), particularly in developing economies. This revealed that women-owned enterprises significantly contribute to job creation, innovation, and local economic development ( Ogundana et al., 2021). Studies have shown that MSEs are essential in fostering entrepreneurship, with women entrepreneurs playing a vital role in creating resilient, community-driven businesses ( Raman et al., 2022). This theme aligns with recent findings by Forster-Holt & Davis (2024), who argue that women’s businesses, especially in small enterprises, are fundamental to achieving sustainable development goals in emerging economies.

**Theme 3: Social impact of women entrepreneurs**

This theme examines the societal contributions of women entrepreneurs, focusing on their role in driving social change and improving community well-being. Keywords like “social”, “evidence”, and “effect” suggest that this topic explores the broader implications of women’s entrepreneurial activities beyond financial outcomes, such as their influence on education, healthcare, poverty alleviation, and social mobility. The theme also highlights an evidence-based approach, indicating the use of empirical studies to measure the social impact of women entrepreneurs. Entrepreneurship provides a transformative platform for women empowerment, particularly in micro enterprises where women have a more significant presence ( Kogut & Mejri, 2021). These findings corroborate earlier research by Brush et al., (2019) on the empowerment potential of women in business.

**Theme 4: Challenges faced by female entrepreneurs**

This theme identifies the barriers and challenges specific to female entrepreneurs, with keywords such as “gender”, “entrepreneurs”, “female”, and “case”. It likely addresses issues such as gender-based discrimination, lack of access to resources, societal biases, and institutional barriers that hinder women’s entrepreneurial efforts. The mention of “case” implies that this theme might include case studies or qualitative analyses to provide detailed accounts of these challenges. This explores the unique barriers faced by women entrepreneurs in MSEs. These barriers include limited access to capital, gender-based discrimination, and a lack of professional networks ( Marlow, 2020; White & Gupta, 2020). The difficulties in scaling women-led businesses often arise from structural inequalities and lack of institutional support, especially in micro and small businesses ( Ahl, 2006). Recent studies ( Agrawal et al., 2023; Bastian et al., 2018; Ghosh, 2024; Vuciterna et al., 2024) emphasize the importance of targeted interventions to address these barriers and promote the growth of women-led enterprises.

### 5.4 Word cloud

**Figure 6.**
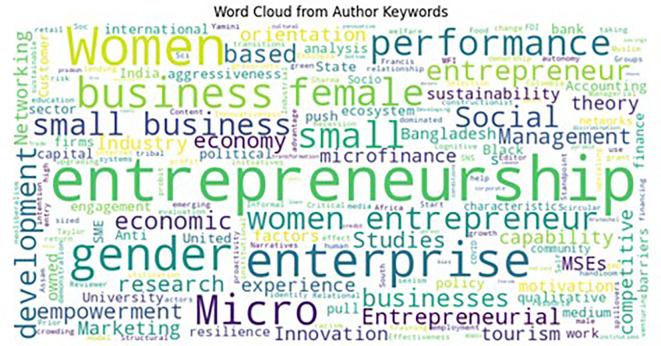
Word Cloud. Source: Author’s own development.

The word cloud represented by Figure 6 illustrates keywords derived from a text corpus, presumably developed using Latent Dirichlet Allocation (LDA), a method for topic modelling. LDA discerns themes or topics within a document corpus, with each topic defined by a distribution of keywords. The word cloud visually emphasizes these keywords, with the size of each word indicating its frequency or significance within the analyzed text. Terms like “entrepreneurship,” “women,” “business,” and “gender” indicate their prominence within the primary subjects of the corpus. This signifies an emphasis on research domains such as entrepreneurship, gender studies, small and micro firms, and performance and development. For example, words like “women,” “female,” and “empowerment” refer to gender-related issues, whereas phrases like “microfinance,” “small business,” and “economic” refer to small-scale business concerns. The word cloud provides a high-level summary of the thematic focus and an intuitive visualization of the LDA results, making it easy to identify dominating topics and key phrases in the text.

## 6. Discussion

This study emphasizes the intricacies of women’s entrepreneurship, concentrating on the obstacles, opportunities, and transformative potential in the Indian setting. Women’s entrepreneurship has become a crucial catalyst for financial autonomy, social empowerment, and regional advancement. The study delineates numerous pivotal domains, encompassing the function of micro and small enterprises (MSEs), the importance of regional differences, and the transformative influence of digital platforms. Notwithstanding advancements in metropolitan locales and fields such as healthcare, arts, and education, systemic obstacles endure. These encompass restricted access to capital, systemic bias, and insufficient support networks, especially in rural and marginalised areas. Digitalization has been a transformative force, equipping women entrepreneurs with tools to surmount old obstacles such as restricted mobility and inadequate market access. Nonetheless, regional disparities persist, with progressive states such as Tamil Nadu and Kerala excelling in women-led firms, but others, especially in the North-East and rural regions, fall behind. The discourse emphasises that these inequities arise from socio-cultural norms, economic infrastructure, and deficiencies in policy execution. These findings underscore the necessity for tailored, region-specific actions to foster a more inclusive entrepreneurial ecosystem.

This study employs Latent Dirichlet Allocation (LDA) to delineate four principal issues about women’s entrepreneurship in India: Women in Entrepreneurship and Gender Roles, Small Business and Women Enterprises, Social Impact of Women Entrepreneurs, and Challenges Faced by Female Entrepreneurs. These themes offer a systematic comprehension of the opportunities and difficulties within the entrepreneurial ecosystem. The theme of Women in Entrepreneurship and Gender Roles underscores the impact of cultural norms and gender dynamics on entrepreneurial involvement, illustrating how patriarchal frameworks frequently constrain women’s chances. The Small Business and Women Enterprises subject indicates that the majority of women engage in micro and small enterprises (MSEs), predominantly within conventional industries, which demonstrates constrained diversification and scalability resulting from structural obstacles such as limited access to capital and professional networks. The Social Impact of Women Entrepreneurs highlights their role in promoting empowerment, generating employment, and tackling community-specific challenges, illustrating the wider societal contributions of women-led enterprises. The issue of Challenges Faced by Female Entrepreneurs highlights systemic barriers, such as financial limitations, gender discrimination, and insufficient digital access, that impede women’s entrepreneurial advancement. LDA’s data-driven research underscores the interaction of these themes, highlighting the necessity for focused measures to foster a more inclusive and supportive entrepreneurial climate.

## 7. Conclusion

This systematic literature review integrates disparate studies on women’s entrepreneurship in India, offering a thorough examination of essential themes like empowerment, digitalization, and regional dynamics. The study highlights the transformative potential of women-led firms, which contribute to economic growth and promote social change and gender equity. Although many industries and locations exhibit encouraging trends, the ongoing problems women encounter, including structural obstacles and societal prejudices, underscore the necessity for further endeavours to establish an inclusive entrepreneurial ecosystem.

This research utilizes Latent Dirichlet Allocation (LDA) modelling to identify theme patterns, providing insights into the interrelation between gender, entrepreneurship, and geographical environments. The findings underscore the unexploited potential of women entrepreneurs, especially in emerging economies, where their efforts are largely unacknowledged. The report ultimately advocates for cooperative initiatives among policymakers, financial institutions, and other stakeholders to overcome obstacles and realize the potential of women entrepreneurs. This study’s LDA analysis reveals four primary themes that underscore the intricacies of women’s entrepreneurship. These themes jointly demonstrate that although women entrepreneurs considerably contribute to economic and social advancement, their potential is hindered by systemic obstacles. Mitigating these hurdles via policies, digital inclusion, and localized initiatives can harness the transformative potential of women-led firms, promoting sustainable and equitable development.

## 8. Future implications

### 8.1 Social future implications

According to the research, women entrepreneurs are critical to promoting social transformation and community development. Micro and small enterprises (MSEs) facilitate women’s attainment of financial autonomy, decision-making authority, and social standing. Women-led enterprises address local social challenges, generate employment, and advance social justice, in addition to providing economic advantages. Digital platforms have enabled women entrepreneurs to surmount mobility and market access challenges. Policymakers and stakeholders must priorities digital literacy initiatives for women, particularly in rural and underserved regions. Commemorating and disseminating the accomplishments of women entrepreneurs might contest societal conventions and motivate forthcoming generations. Improving community-based education, healthcare, and poverty reduction initiatives will amplify the impact of women entrepreneurs and foster a more inclusive and fair society.

### 8.2 Managerial future implications

This research suggests that managerial support for women entrepreneurs necessitates specialized strategies. Programs focused on skill development in management, finance, and technology may assist women in overcoming corporate issues. Women-led firms can enhance market access, operational efficiency, and competitiveness through the utilization of digital tools. Policymakers should implement region-specific strategies to support women entrepreneurs by addressing local socio-cultural and economic factors. Establishing collaborative ecosystems with enterprises, governments, and industry experts can facilitate mentorship, resources, and networking opportunities for innovation and development. These programs assist women-led firms in achieving their maximum potential and fostering sustainable economic development.

## 9. Limitations

The study acknowledges certain limitations that may guide future research. The examination of Indian women MSE entrepreneurs restricts its relevance to other nations and industries. Extensive cross-cultural study is required to validate and enhance these findings. Secondly, existing literature may neglect emerging entrepreneurial trends and innovations. A comprehensive understanding would emerge from the collection of primary data and the analysis of real-time case studies. Third, although the thematic analysis provides qualitative insights, the absence of quantitative data constrains the assessment of the direct impacts of policies and programs on women entrepreneurs. Subsequent research should employ hybrid methodologies for enhanced insights. The study overlooks intersectional elements such as caste, religion, and socioeconomic status, which significantly influence Indian entrepreneurship. Intersectional research may reveal more intricate difficulties and opportunities, enhancing our comprehension of women’s entrepreneurship. Addressing these limitations will lay the framework for inclusive entrepreneurship policies and initiatives.

## Ethics and consent

Ethical approval and consent were not required.

## Data Availability

Zenodo: Mapping the landscape of women entrepreneurs in micro and small enterprises: Trends, themes, and insights through systematic review and text mining. doi.org/10.5281/zenodo.15014239 ( Kumar. M., 2025c) The project contains the following underlying data:
1. WE&MSEs.csv - Bibliometric Data Data are available under the terms of the Creative Commons Attribution 4.0 International license (CC-BY 4.0). Zenodo: Mapping the landscape of women entrepreneurs in micro and small enterprises: Trends, themes, and insights through systematic review and text mining. /doi.org/10.5281/zenodo.15072788 ( Kumar. M., 2025b) The project contains the following Extended data: Table of Authors, Title, Year, Source title, Topic Distribution and Findings using LDA
1. Extended Data.docx - The project contains the following data: Table of Authors, Title, Year, Source title, Topic Distribution and Findings using LDA Data are available under the terms of the Creative Commons Attribution 4.0 International license (CC-BY 4.0). *Zenodo: PRISMA Checklist for* Mapping the landscape of women entrepreneurs in micro and small enterprises: Trends, themes, and insights through systematic review and text mining, doi.org/10.5281/zenodo.15077967 ( Kumar. M., 2025a) The project contains the following-
1. PRISMA Flowchart 2. PRISMA Checklist
