# Supplementary material for: Mapping the landscape of women entrepreneurs in micro and small enterprises: Trends, themes, and insights through systematic review and text mining
Source: F1000Res. 2025 Apr 10;14:422. [Version 1] doi: 10.12688/f1000research.163030.1 (PMC12153045; doi:10.12688/f1000research.163030.1)
Supplement: Peer review history

# Reviewer report: Reviewer response for version 1

**DOI:** 10.5256/f1000research.179322.r378117

**Published:** 2025-05-03

**Authors:** Sudhir Rana

**Affiliations:** 1 Gulf Medical University, Ajman, United Arab Emirates

Consistency in Terminology (MSMEs vs. MSEs)

 Throughout the paper, both MSEs and MSMEs are used interchangeably. Consider standardizing this usage. If both are necessary, clarify the distinction early (perhaps in Section 1 or 4).

 2. Reinforce Link Between Literature Review and Research Questions. Section 2 (Literature Review) can more clearly connect back to the three Research Questions in Section 3. Right now, the bridge is implied but not explicit. A paragraph that highlights

 how the literature gaps inform RQ1–RQ3 would help.

 3. Tighten the Writing in the Introduction. The opening section is informative but slightly verbose. Condensing redundant phrases (e.g.,

 “economic growth and social transformation” repeated multiple times) can make the intro more impactful.

 4. Figure Captions Need Better Descriptors. Figures 2–6 are labelled generically (“Source: Author’s development”), but it would be

 helpful to provide clearer captions. For example: “Figure 3: Annual number of articles published on women entrepreneurship in MSEs (2000–2024).”

 It is important that review methodology is updated with the latest arguments, latest styles of reviewing, and holistic review techniques. I am sure the latest paper below would be helpful to you. In the paper below look at the annexure given to understand what is required in which section.

 Being a researcher on the literature review domain, I suggest reading the following volume. The papers published in this volume are useful in understanding how to write an impactful review paper and crafting the methodology section:

 Ref: 1

https://www.emerald.com/insight/publication/doi/10.1108/S2754-5865202402

Is the work clearly and accurately presented and does it cite the current literature?

Yes

If applicable, is the statistical analysis and its interpretation appropriate?

Yes

Are all the source data underlying the results available to ensure full reproducibility?

Yes

Is the study design appropriate and is the work technically sound?

Yes

Are the conclusions drawn adequately supported by the results?

Yes

Are sufficient details of methods and analysis provided to allow replication by others?

Yes

Reviewer Expertise:

Marketing and Strategy

I confirm that I have read this submission and believe that I have an appropriate level of expertise to confirm that it is of an acceptable scientific standard, however I have significant reservations, as outlined above.

## References

[1] : Parameters and Decision Elements of Writing Effective Literature Review Papers: Empirical Evidence From Multiple Stakeholders on POWER Framework. 10.1108/S2754-586520230000002001 1-25 10.1108/S2754-586520230000002001

# Reviewer report: Reviewer response for version 1

**DOI:** 10.5256/f1000research.179322.r378122

**Published:** 2025-04-30

**Authors:** Narayanage Jayantha Dewasiri

**Affiliations:** 1 Sabaragamuwa University of Sri Lanka, Belihuloya, Sri Lanka

1. Expand on Limitations Related to Text Mining

 In Section 9 (Limitations), while the geographical and methodological limitations are well-

 stated, there is no mention of potential bias in LDA modeling (e.g., reliance on abstracts over

 full text). A sentence acknowledging this would show methodological awareness.

 2. Enhance Discussion on Practical Implications

 The “Managerial Future Implications” section could better distinguish between general

 suggestions and evidence-based insights from your findings. For example, if digitalization

 emerged as a theme, specify what kind of digital tools or interventions were most impactful

 in the reviewed studies.

 3. Improve Abstract Clarity and Structure

 The abstract is conceptually strong but would benefit from clearer paragraph separation for

 Background, Methods, Results, and Conclusion (even if not using explicit headers). This helps

 readers quickly grasp the scope and contributions of the paper.

Is the work clearly and accurately presented and does it cite the current literature?

Yes

If applicable, is the statistical analysis and its interpretation appropriate?

Yes

Are all the source data underlying the results available to ensure full reproducibility?

Yes

Is the study design appropriate and is the work technically sound?

Yes

Are the conclusions drawn adequately supported by the results?

Yes

Are sufficient details of methods and analysis provided to allow replication by others?

Yes

Reviewer Expertise:

Entrepreneurship

I confirm that I have read this submission and believe that I have an appropriate level of expertise to confirm that it is of an acceptable scientific standard.

# Reviewer report: Reviewer response for version 1

**DOI:** 10.5256/f1000research.179322.r378114

**Published:** 2025-04-23

**Authors:** Sucheta Agarwal

**Affiliations:** 1 GLA University, Mathura, India

1. Kindly add the "Policy implications" also

 2. If deemed appropriate, the following papers may be referred to for enriching the manuscript:

 a. Role of women entrepreneurs and NGOs in promoting entrepreneurship: case studies from Uttarakhand, India

 b. An exploratory study on the development of women entrepreneurs: Indian cases

 c. A qualitative approach towards crucial factors for sustainable development of women social entrepreneurship: Indian cases

 d. Does growth of ventures depend on competencies?: selected cases from India

Is the work clearly and accurately presented and does it cite the current literature?

Yes

If applicable, is the statistical analysis and its interpretation appropriate?

Yes

Are all the source data underlying the results available to ensure full reproducibility?

Yes

Is the study design appropriate and is the work technically sound?

Yes

Are the conclusions drawn adequately supported by the results?

Partly

Are sufficient details of methods and analysis provided to allow replication by others?

Yes

Reviewer Expertise:

Women entrepreneurship

I confirm that I have read this submission and believe that I have an appropriate level of expertise to confirm that it is of an acceptable scientific standard.
